# Direct Pro-Inflammatory Effects of Prorenin on Microglia

**DOI:** 10.1371/journal.pone.0092937

**Published:** 2014-10-10

**Authors:** Peng Shi, Justin L. Grobe, Fiona A. Desland, Guannan Zhou, Xiao Z. Shen, Zhiying Shan, Meng Liu, Mohan K. Raizada, Colin Sumners

**Affiliations:** 1 Department of Physiology and Functional Genomics, College of Medicine, University of Florida, Gainesville, Florida, United States of America; 2 Department of Neurology, Cedars-Sinai Medical Center, Los Angeles, California, United States of America; 3 Department of Pharmacology, Roy J & Lucille A. Carver College of Medicine, University of Iowa, Iowa City, Iowa, United States of America; 4 Department of Biomedical Science, Cedars-Sinai Medical Center, Los Angeles, California, United States of America; 5 Department of Kinesiology and Integrative Physiology, Michigan Technological University, Houghton, Michigan, United States of America; Indiana School of Medicine, United States of America

## Abstract

Neuroinflammation has been implicated in hypertension, and microglia have been proposed to play an important role in the progression of this disease. Here, we have studied whether microglia are activated within cardiovascular regulatory area(s) of the brain during hypertension, especially in high blood pressure that is associated with chronic activation of the renin-angiotensin-system. In addition, we determined whether prorenin, an essential component of the renin-angiotensin-system, exerts *direct* pro-inflammatory effects on these microglia. Our data indicate that two rodent models which display neurogenic hypertension and over activation of the renin-angiotensin-system in the brain (sRA mice and spontaneously hypertensive rats) exhibit microglial activation, and increased levels of pro-inflammatory cytokines, in the paraventricular nucleus of the hypothalamus, an area crucial for regulation of sympathetic outflow. Further, the renin-angiotensin-system component prorenin elicits *direct* activation of hypothalamic microglia in culture and induction of pro-inflammatory mechanisms in these cells, effects that involve prorenin receptor-induced NFκB activation. In addition, the prorenin-elicited increases in cytokine expression were fully abolished by microglial inhibitor minocycline, and were potentiated by pre-treatment of cells with angiotensin II. Taken together with our previous data which indicate that pro-inflammatory processes in the paraventricular nucleus are involved in the hypertensive action of renin-angiotensin-system, the novel discovery that prorenin exerts direct stimulatory effects on microglial activation and pro-inflammatory cytokine production provides support for the idea that renin-angiotensin-system -induced neurogenic hypertension is not restricted to actions of angiotensin II alone.

## Introduction

Hypertension is a global health problem, and ∼20 to 30% of hypertensive patients are resistant to the available anti-hypertensive medications [1], [2]. It is generally accepted that the uncontrolled, resistant hypertension is primarily neurogenic in origin, involving chronic over activity of the sympathetic nervous system that initiates and sustains high blood pressure [3]–[5]. It is well known that angiotensin II (Ang II) acting via its type 1 receptor (AT1R) within the paraventricular nucleus of the hypothalamus (PVN), a brain site that plays a crucial role in regulating sympathetic outflow [6], [7], is a major contributor to this chronic sympathoexcitation [8]. In addition to this Ang II/AT1R mechanism it is now evident that another member of the renin-angiotensin system (RAS), prorenin, and its receptor PRR [9] play a role in the central control of neurogenic hypertension. For example, we have shown that down-regulation of PRR in the supraoptic nucleus via viral-mediated transduction significantly attenuates blood pressure development in spontaneous hypertensive rats (SHR) [10]. Furthermore, PRR levels are significantly higher in the PVN of human renin-angiotensinogen double-transgenic hypertensive mice, another model of neurogenic hypertension, and knockdown of PRR in the brains of these animals significantly decreased blood pressure and sympathetic vasomotor tone [11]. A more recent study further confirms the contribution of PRR in hypertension development showing that neuron-specific knockdown of PRR lowers Ang II formation and blood pressure in the deoxycorticosterone acetate-salt mouse model of hypertension [12]–[14].

Accumulating evidence suggests that neuroinflammatory processes make a significant contribution to the pathological processes underlying sustained high blood pressure [11], [12]. For example, in previous studies we demonstrated that viral vector-mediated increases in the expression of the anti-inflammatory cytokine interleukin-10 (IL-10) specifically within the PVN significantly reduced blood pressure in rats made hypertensive by chronic systemic infusion of angiotensin II (Ang II) [15]. In the same set of studies we demonstrated that Ang II induced hypertension was significantly decreased by central (intracerebroventricular; ICV) infusion of minocycline, a tetracycline antibiotic that inhibits activation of microglia, the primary resident immune cells in the brain. Furthermore, this minocycline treatment abolished the increases in mRNAs for pro-inflammatory cytokines (IL-1β; IL-6; tumor necrosis factorα {TNFα}) and the decrease in IL-10 mRNA in the PVN elicited by Ang II infusion [15]. It has also been demonstrated that Ang II-induced hypertension is dependent upon activation of the inflammatory factor nuclear factor kappa B (NFκB) in the PVN [16] and direct injection of IL-1β into the PVN or via the intracerebroventricular route increases mean arterial pressure [12], [15], [16]. Our recent studies show that prorenin also induces the increases in pro-inflammatory cytokine expression in the nucleus tractus solitarius (NTS), an effect that involves activation of the nuclear factor kappa B (NFκB) complex [17]. Down-regulation of PRR mediated by viral transfection in the supraoptic nucleus (SON) significantly slows down high blood pressure accompanied by decreased inflammatory markers [10]. Collectively these studies support the idea that neuroinflammatory processes within the PVN are involved in the hypertensive action of the RAS. It should also be pointed out that the inflammatory mechanisms associated with neurogenic hypertension are not restricted to the PVN in the brain, as studies indicate that the nucleus tractus solitarius, another important cardiovascular control center, exhibits an inflammatory state in spontaneously hypertensive rats (SHR), an animal model of this disease [18].

Thus, the idea we are promoting is that a low (but persistent) level of microglial activation in the PVN occurs via brain RAS activation in neurogenic hypertension. Questions raised by the above investigations are whether the inflammation observed in the PVN during neurogenic hypertension is due to over activation of the brain (rather than peripheral) RAS, and whether the pro-inflammatory actions of prorenin include *direct* effects at microglia. To address these questions in the present study we have first assessed the levels of microglial activation and of cytokine expression in the PVN of both double transgenic mice (sRA mice) that have selective over activation of the brain RAS (genetic overexpression of human renin and angiotensinogen controlled by specific promoters in the brain) [19], and SHR. Considering recent findings that the PRR in the brain contributes to the pathogenesis of hypertension [20], we tested whether prorenin exerts *direct* pro-inflammatory actions via microglia. We have utilized cell culture systems, a mouse microglial cell line (N-9) and microglia isolated from rat hypothalamus, to determine whether prorenin has direct actions on these cells. The findings made in this study support the general idea that brain RAS-mediated activation of pro-inflammatory mechanisms is an important contributor to neurogenic hypertension.

## Methods

### Animals and materials

Male spontaneously hypertensive rats (SHR), Wistar Kyoto (WKY) rats (8-week-old), and timed pregnant (E13–15) Sprague-Dawley (SD), SHR and WKY rats were obtained from Charles River Farms (Wilmington, MA). Double transgenic sRA mice [19] were bred and housed at the University of Iowa, and were used at 12–21 weeks of age. Male double-transgenic sRA mice were generated as described previously by cross-mating C57BL/6J mice that express human renin via the synapsin promoter (sR mice) with mice expressing human angiotensinogen with its own promoter (A mice). Littermates with either expression of human angiotensinogen or renin were used as controls. These mice were 12–21 weeks of age, matched exactly with the sRA mice. All rats and mice were housed individually in shoebox style forced-air cages, with access to tap water and food *ad libitum and* with a 12∶12 hr light/dark cycle. The Institutional Animal Care and Use Committees of the University of Florida and the University of Iowa approved all protocols for animal use. The reagents used in this study are as follows: Human Prorenin was purchased from Innovative Research (IHPREN, Novi, MI); Iba1 antibody was from Wako (01–1974, Richmond, VA) and Abcam (ab5076, San Francisco, CA); prorenin receptor antibody from Abcam (ab64957); anti-CD11b from Abcam (ab52478); anti-β actin from Sigma (A2228, St Louis, MO); real-time primers from Applied Biosystems (Grand Island, NY); and antibodies for flow cytometry and ELISA from eBioscience (San Diego, CA).

### Microglial cultures

N-9 mouse microglial cells (gift from Dr. Gerry Shaw, University of Florida) were cultured in Dulbecco's Modified Eagles Medium (DMEM) containing 10% fetal bovine serum (FBS) and an antibiotic cocktail (10,000 IU/ml Penicillin and 10,000 µg/ml Streptomycin; Cellgro, Manassas, VA).

Primary microglial cells were prepared from newborn SD, SHR and WKY rat pups based on published studies [21]. Meninges and choroid plexus membranes were removed from brains, and a hypothalamic block containing the PVN was dissected and minced with small scissors. The dissected tissues were further dissociated by filtering through a 100 µm pore nylon mesh (BD, Franklin Lakes, NJ), followed by centrifuging three times (300×g; 5 min/spin) at room temperature. After the third centrifugation the pellets were re-suspended in DMEM containing 10% FBS and an antibiotic cocktail (1%) as above. The microglial cells were seeded onto Poly-D-lysine coated 100-mm dishes at a density of 1−5×10^5^/ml, and incubated with 95% O_2_/5% CO_2_ at 37°C for 7 days without changing the medium. After this time period, floating microglia in the culture medium were collected and transferred to 6-well plates for 3 hr, and the original dishes were fed with fresh medium. Microglia were collected in this way at weekly intervals. Isolated cells were pure microglia based on the co-localization of Iba1 immunoreactivity with DAPI fluorescence. After attachment in the new plates, microglia were fed with fresh DMEM/10% FBS/1% antibiotic cocktail, and incubated for another 2–3 days before each treatment.

### Iba1 immunohistochemistry

PVN: Immunoreactivity for the microglial marker Iba1 in the PVN of mice (sRA and wild type) or rats (SHR and WKY) was assessed as follows. Animals were deeply anesthetized with 5% isoflurane mixed with O_2_, and perfused transcardially with heparinized saline followed by 4% paraformaldehyde. Brains were removed and coronal sections (20 µm) cut through the hypothalamus to capture the PVN. Sections were incubated with a polyclonal rabbit anti-Iba1 antibody (1∶500, Wako) followed by incubation with a biotinylated polyclonal rabbit anti-rabbit IgG (1∶200). After additional rinsing, sections were incubated with avidin-peroxide conjugate containing 0.04% 3,3′-diaminobenzidine hydrochloride for 10 min. Sections were mounted on glass slides with cover slips using Vectashield mounting medium with DAPI (Vector Labs, Burlingame, CA).

Microglial cell cultures: Cultured N-9 cells or primary microglia were washed with PBS and then fixed for 10 min with ice cold 0.3% Triton X-100 for 20 min to improve antibody penetration. Goat serum (5%) in PBS was added to the dish for 30 min at 37°C to reduce nonspecific binding, followed by an additional wash with PBS. Immunocytochemistry was performed using either a rabbit polyclonal antibody against Iba1 (1∶2000) or a goat polyclonal antibody against the PRR (1∶500, Abcam). This was followed by incubation with respective secondary antibodies, Alexa Fluor 488 goat anti-rabbit IgG (1∶2000) or Alexa Fluor 594 horse anti-goat IgG (1∶2000) (Molecular Probes, Eugene, OR). PRR and Iba1 immunoreactivities were detected using an Olympus BX41 fluorescence microscope.

### Real-time RT-PCR

For the analysis of mRNA levels, brain tissues or microglial cells (primary microglia or N-9 cells) were processed as described previously [15]. In brief, RNA was extracted using RNeasy Mini Plus kits (Qiagen, Valencia, CA). Purified RNA (200 ng) was reverse transcribed using a high-capacity iScript cDNA synthesis kit (Bio-Rad, Hercules, CA). The expression levels of PRR, IL-1β, IL-10, TNFα, 18 s and GAPDH mRNAs were analyzed via quantitative real-time RT-PCR using an ABI OneStep Plus machine (Applied Biosystems). 18 s ribosomal RNA or GAPDH was used as internal controls.

### TNFα ELISA

Anteroventral 3^rd^ ventricle (AV3V), PVN, NTS and rostral ventrolateral medulla (RVLM) tissues from control and sRA mice were dissected and homogenized in RIPA buffer (Thermo, Rockford, IL). TNFα was measured using an ELISA kit according to the manufacturer's instructions (eBioscience) and analyzed by Fluostar Omega microplate reader (BMG Labtech, Oretenberg, Germany).

### Western Blot for CD11b in cultured microglia

The collected microglial samples were homogenized with Laemmli Sample buffer (BioRad), loaded onto 4–15% gradient Criterion gels (Bio-Rad) followed by electrophoretic transfer to Nitrocellulose membranes (Bio-Rad) using a standard protocol. Samples were normalized to 1 µg/ml and the loading volume was 20 µl/well. The membranes were incubated with rabbit polyclonal CD11b (2 µg/ml, Abcam) and mouse anti-β-actin (1∶5000, Sigma) over night at 4°C, followed by reactions with respective goat anti-rabbit (1∶5000) and –mouse (1∶5000) (Sigma) peroxidase conjugates. The protein bands for CD11b and β-actin were visualized by chemiluminescence reagents (Amersham, Piscataway, NJ) and quantified by Image J software.

### Flow Cytometry

To measure cytokine expression, mouse N-9 cells were cultured with 5 µg/ml brefeldin A (eBioscience), which prevents protein transportation and leads to cytokine accumulation intracellularly. Cells were surface-stained with FITC-conjugated anti-CD11b (BioLegend, San Diego, CA) followed by intracellular staining of APC-conjugated anti-TNFα (eBioscience) and anti-IL-1β (eBioscience) with fixation and permeabilization buffer (eBioscience). The stained samples were analyzed on a Beckman Coulter CyAn ADP and data were analyzed with FlowJo software.

### Calculation of Microglial Fractional Area and Data Analyses

The activation of PVN microglia, identified by Iba1 immunoreactivity, was analyzed by measuring the fractional area of the cells using Image J software (NIH). The fractional area of each image was analyzed based on the ratio of the calculated area of Iba1-positive staining to the entire image as described previously [15]. The PVN sections that were analyzed covered the entire PVN (Rat: Bregma −1.6 to −2.2 mm; Mouse: Bregma −0.82 to −1.06 mm) at 40× magnification. The number of microglia in a 0.2×0.2 mm^2^ area was counted from 3 different levels covering anterior, medial and posterior of PVN in in each animal. Morphological analysis and quantification of microglia were performed within the PVN using a fluorescence microscope (Olympus BX41). Five animals from each group were used for this analysis, and 15–20 images were taken for each animal.

All N-9 cell and primary microglial culture experiments were performed in quadruplicate wells and repeated at least three times. Data are expressed as mean ± SEM. Statistical significance was evaluated with the use of Prism software (v5.0, GraphPad).

## Results

### Pro-inflammatory cytokine expression in central cardiovascular regulatory nuclei in hypertensive animals

**Figure 1:**
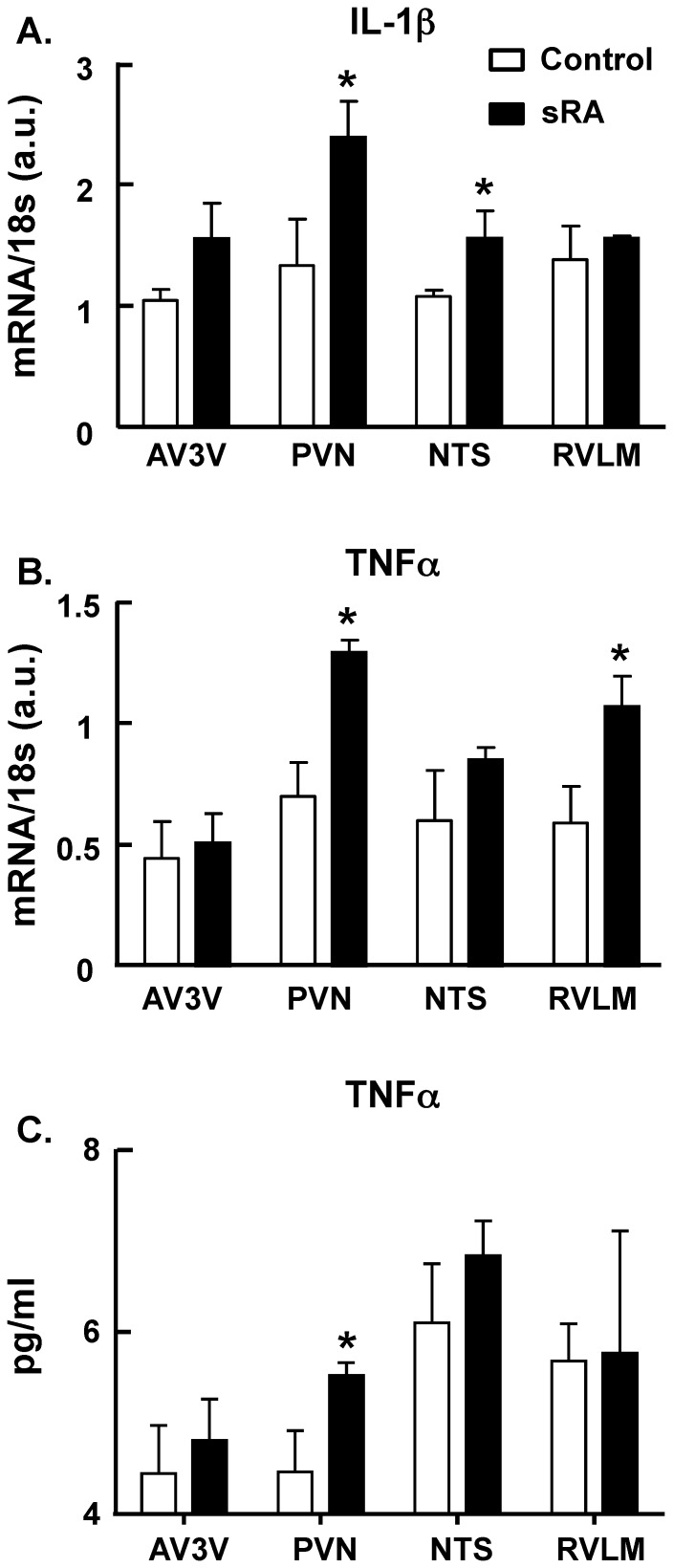
Levels of pro-inflammatory cytokines within central cardiovascular control centers of control and sRA mice. Levels of IL-1β (**A**) and TNFα (**B**) mRNAs and TNFα protein (**C**) were measured in the AV3V, PVN, NTS and RVLM of control and sRA mice as described in the Methods. Data are mean ± SEM., from 6 control and 6 sRA mice for the mRNA and TNFα protein measurements. * P<0.05 vs. respective control.

To characterize neuroinflammation in hypertension, we examined in hypertensive animals the expression of two major pro-inflammatory cytokines in different brain regions related to cardiovascular function, the AV3V region, the PVN, the NTS and the RVLM. The above brain regions were dissected from hypertensive sRA and normotensive control mice. We found that the expression of mRNAs for both IL-1β and TNFα (Figure 1) were significantly increased in the PVN of hypertensive sRA mice compared to the normotensive controls, which is consistent with our previous study [15]. In addition, the mRNA levels of IL-1β in the NTS and TNFα in the RVLM were significantly greater in the sRA mice when compared to control mice. In further experiments, we examined TNFα protein expression in the above four brain regions of normotensive and sRA mice. The data demonstrated that TNFα protein levels were significantly greater only in the PVN of sRA mice compared to the controls. Collectively, these data suggest that the hypothalamic PVN of hypertensive mice is a primary site of neuroinflammation amongst the central cardiovascular control centers.

### Animal models of neurogenic hypertension display increased microglial activation and RAS expression in the PVN

**Figure 2:**
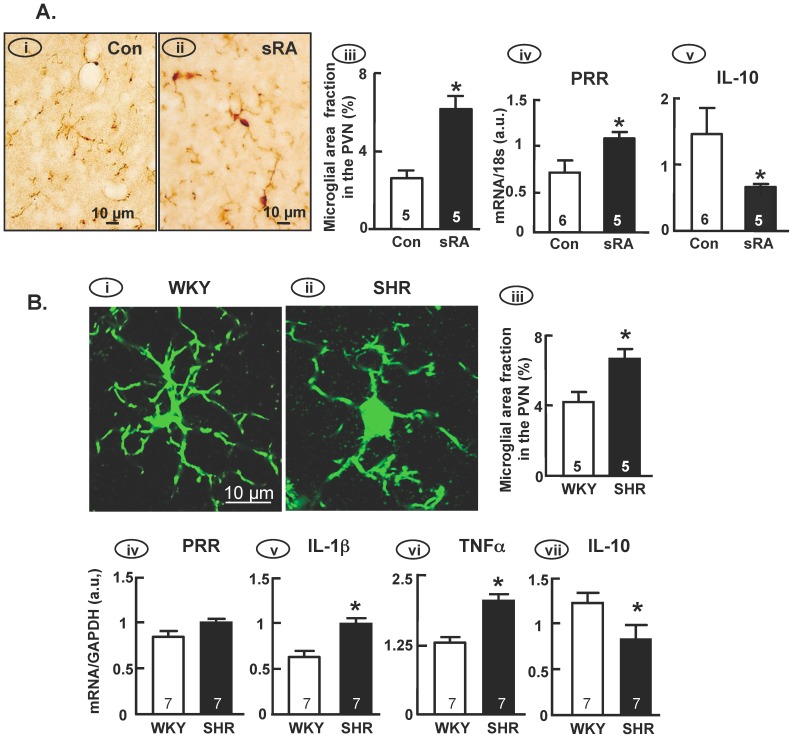
Microglial activation in the PVN of sRA mice and SHR. (**A**) *Control vs. sRA mice*: Panels (i) and (ii) are representative bright field micrographs showing Iba1 immunoreactivity in the PVN of control and sRA mice. Panels (iii-v) are bar graphs comparing the microglial fractional area (iii), and the levels of PRR (iv) and IL-10 (v) mRNAs in the PVN of control and sRA mice. Data are means ± SEM from the numbers of mice indicated within the bars. *P<0.05 vs. respective control. (**B**) *SHR vs. WKY rats*: Panels (i) and (ii) are representative bright field micrographs showing Iba1 immunoreactivity in the PVN of WKY rats and SHR. Panels (iii-vii) are bar graphs comparing the microglial fractional area (iii), and the levels of PRR (iv), IL-1β v), TNFα (vi) and IL-10 (vii) mRNAs in the PVN from both strains. Data are means ± SEM from the numbers of mice indicated within the bars. *P<0.05 vs. respective control.

In this set of studies we determined the profiles of microglial activation and RAS expression in the PVN of sRA mice and SHR versus their respective control animals. Both of these animal models of neurogenic hypertension exhibit enhanced RAS activity in the CNS, increased sympathetic outflow and resting arterial blood pressure [16], [22]. Iba1 immunostaining revealed that microglia within the PVN of control mice exist primarily in the “resting” state, as illustrated by small somas and long branched processes (Figure 2Ai). In contrast, the enlarged soma and shorter processes of PVN microglia in sRA mice suggest that these cells are activated (Figure 2Aii). Quantification of microglial activation was performed using the fractional area method, counting the area occupied by microglia based on Iba1 immunoreactivity. The microglial fractional area was significantly increased in the PVN of sRA mice compared to control mice (Figure 2Aiii). In addition, real-time PCR analyses revealed that sRA mice exhibit increased levels of PRR mRNA and a decreased level of mRNA for the anti-inflammatory cytokine IL-10, in the PVN compared to controls (Figure 2Aiv-v). The same strategy was used to examine microglial activation in SHR and WKY rats (Figure 2B). Microglia within the PVN of SHR, identified by Iba1 immunoreactivity, exhibited increased activation based on a preponderance of cells exhibiting a larger soma, compared with a largely ramified appearance and a smaller soma in WKY rat PVN (Figure 2Bi-iii). Although there was no obvious difference in PRR mRNA levels in the PVN of SHR and WKY rats (Figure 2Biv), the levels of IL-1β and TNFαmRNAs were significantly increased, while IL-10 mRNA was significantly decreased, in the PVN of SHR compared to WKY rats (Figure 2Biv-vii).

### Prorenin activates microglial cells in culture

**Figure 3:**
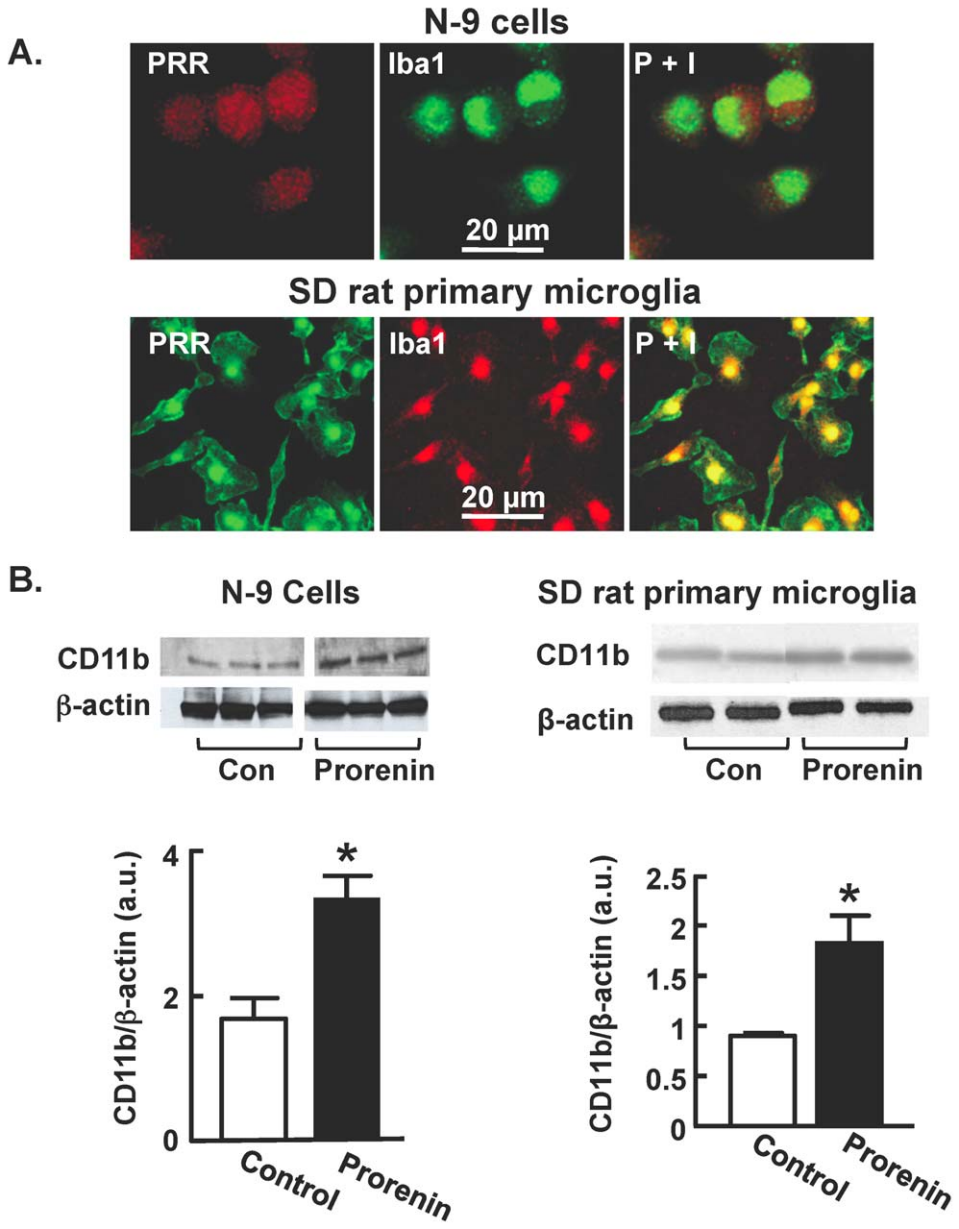
PRR expression and function in microglia. (**A**) (i) Representative fluorescence micrographs showing immunoreactive PRR (P) and Iba1 co-localized (P+I) in mouse N-9 microglial cells and SD rat primary microglia. (**B**) CD11b protein expression was analyzed in N-9 cells and SD rat primary microglia by western blotting following 24 hr treatment with either control media (DMEM) or prorenin (20 nmol/L). *Top*: Representative immunoblots showing CD11b and β-actin (loading control) protein bands under each treatment condition; *Bottom*: Bar graphs are band density ratios of CD11b protein normalized against β-actin. Data are means ± SEM, n = 6 experiments.

Since sRA mice and SHR exhibit increased activation of microglia within the PVN, along with increased levels of mRNAs for PRR (only in sRA mice) at this site (Figure 2Aiv), we investigated whether there were any *direct* actions of prorenin on microglial cell activation. For these experiments we established *in vitro* cultures of the mouse-derived microglial N-9 cell line and also primary microglia dissociated from the hypothalamus of newborn SD or WKY rats, or SHR. Immunocytochemistry clearly demonstrated PRR immunoreactivity in the soma of Iba1-positive N-9 cells and SD rat primary microglia (Figure 3A), with similar staining obtained in WKY rat and SHR primary microglia (data not shown). Incubation of either N-9 cells or SD rat primary microglia with human recombinant prorenin (20 nmol/L) for 24 hr elicited significant increases in the levels of CD11b protein, a marker expressed by activated microglia [18] (Figure 3B). These data suggest that prorenin can *directly* activate microglia.

### Prorenin elicits increases in cytokine production in microglia

**Figure 4:**
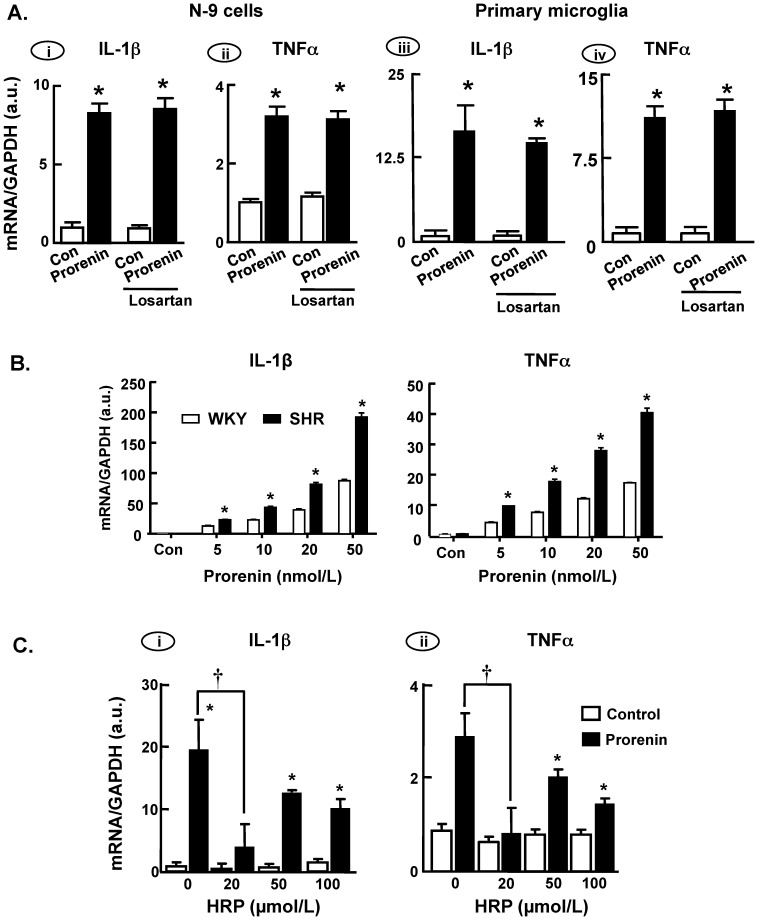
Prorenin increases pro-inflammatory cytokine levels in microglia. (**A**) Mouse N-9 cells (i–ii) and SD rat primary microglial cells (iii–iv) were treated with prorenin (20 nmol/L) in the absence or presence of losartan (1 µmol/L) for 24 hr, followed by analysis of IL-1β and TNFα mRNA levels as detailed in the Methods. Data are means ± SEM, n = 3 experiments. * P<0.05 vs. respective control. (**B**) Levels of IL-1β and TNFα mRNAs were measured after 6 hr treatment with prorenin (5–50 nmol/L) in cultured microglial cells prepared from WKY rats and SHR. Data are means ± SEM, n = 4–5 experiments. All prorenin-treated groups were significantly different vs. their respective controls (P<0.05). * P<0.05 vs. respective WKY rat group. (**C**) Levels of IL-1β and TNFα mRNAs were analyzed after 24 hr treatment of SD rat primary microglial cells with control solution (DMEM) or prorenin (20 nmol/L) in the absence or presence of the indicated concentrations of HRP. Data are means ± SEM from n = 3 experiments. * P<0.05 vs. respective control; **†** P<0.05 vs. prorenin-alone group.

Prorenin is well known to increase pro-inflammatory cytokine production in tissues such as the eye [23], the kidney [24] and the vasculature [25], effects mediated by PRR. To further evaluate the direct effects of prorenin on microglial activation, we tested its effects on pro-inflammatory cytokine levels in cultured microglia. Incubation of N-9 cells or SD rat primary microglia with human recombinant prorenin (20 nmol/L; 3 or 24 hr) elicited significant increases in IL-1β and TNFα mRNA levels, with similar effects at each time point. Shown in Figure 4A are the data from 24 hr. These effects of prorenin in N-9 cells and primary microglia were concentration dependent, with a significant increase obtained at 5 nmol/L. This is illustrated by the data presented in Figure 4B, which shows the concentration-dependency of prorenin's effects in WKY rat and SHR primary microglia. Similar profiles were obtained in N-9 cells and SD rat microglia (data not shown). It was also apparent that these effects of prorenin were significantly greater in SHR than in WKY cells (Figure 4B) and SD rat cells (data not shown). It is also important to point out that these stimulatory effects of human prorenin on IL-1β and TNFα mRNA levels in microglia were not affected by co-treatment with polymixin B (10 ng/mL), excluding the possibility that they were due to lipopolysaccharide contamination of the prorenin.

The stimulatory effects of prorenin on IL-1β and TNFα mRNA levels were unaltered by co-treatment of N-9 or SD rat primary microglial cells with the AT1R blocker losartan (1 µmol/L) (Figure 4A). In contrast, as shown in Figure 4C, the stimulatory effects of prorenin on IL-1β and TNFα mRNA levels in SD rat microglia were abolished by co-treatment of cells with the putative PRR blocker HRP (Handle Region Peptide, 20 µmol/L) [26]. Higher concentrations of HRP (50 and 100 µmol/L) also reduced the effects of prorenin on IL-1β and TNFα mRNA levels, but the effects were not significant. Collectively, these data suggest that the observed effects of prorenin are PRR-dependent.

**Figure 5:**
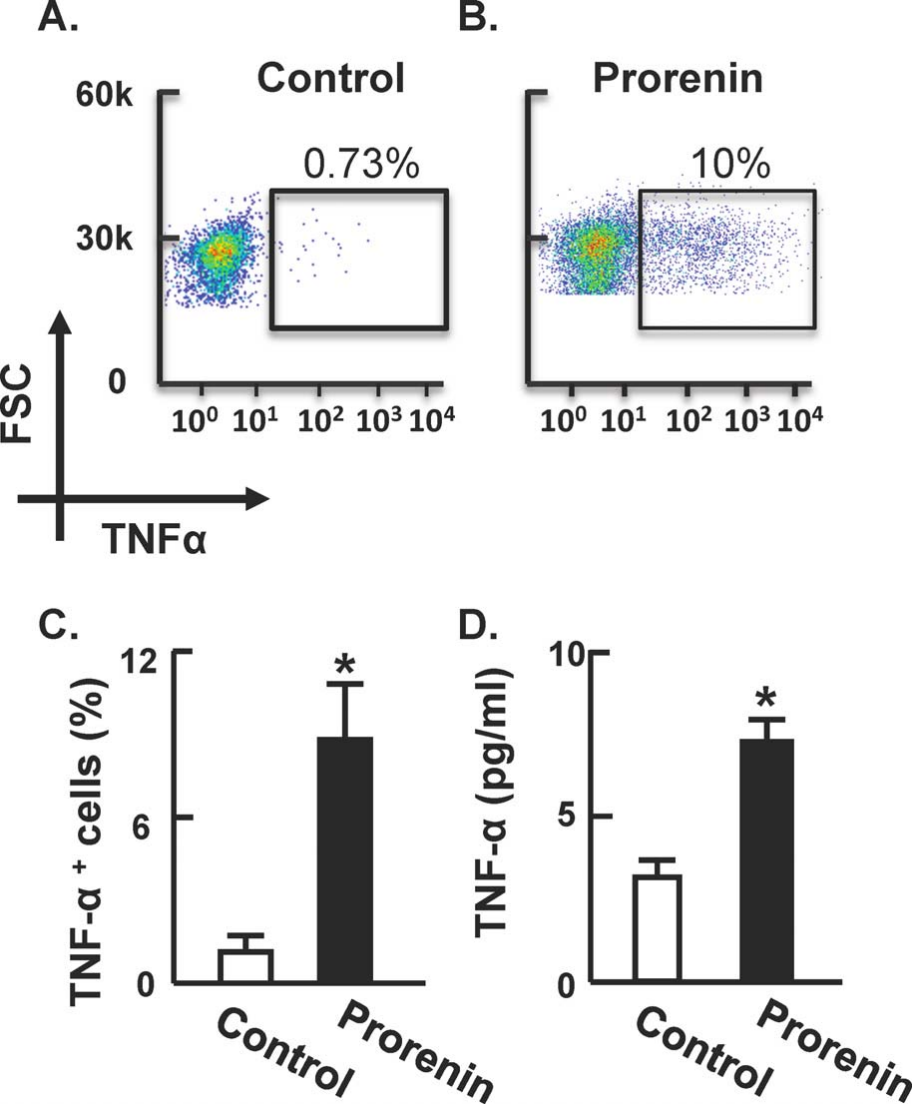
Prorenin increases TNFα levels in N-9 microglial cells. N-9 cells were treated with control medium or prorenin (20 nmol/L) for 6 hr, followed by analysis of TNFα-positive cells using flow cytometry (**A–C**) and released TNFα by ELISA assay (**D**). FSC: forward scatter. Data are means ± SEM from n = 3 experiments. * P<0.05 vs. control.

Considering these effects of prorenin on pro-inflammatory cytokine mRNA levels in cultured microglia, we examined whether prorenin was able to alter the expression of TNFα protein. Flow cytometry analyses revealed that treatment of N-9 cells with prorenin (20 nmol/L; 6 hr) elicited a highly significant increase in TNFα protein (Figure 5A–C). Furthermore, similar treatment of N-9 cells with prorenin (20 nmol/L) elicited a significant increase in TNFα protein levels in the growth media, based on ELISA analyses (Figure 5D).

### Prorenin elicited NFκb activation in microglia

**Figure 6:**
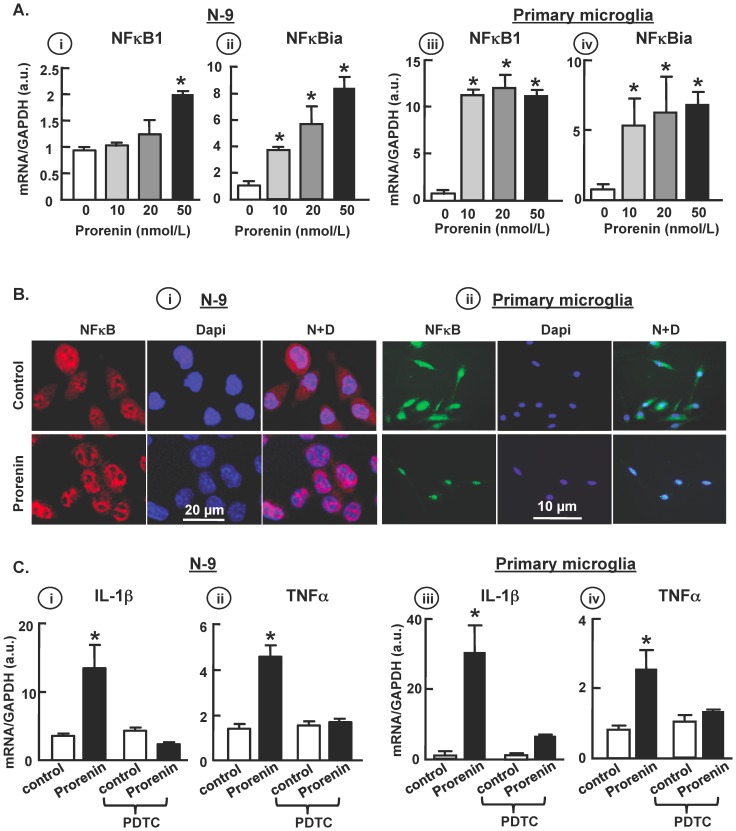
NFκB activation mediated prorenin-induced cytokine production. (**A**) N-9 (i–ii) and primary microglial cells (iii–iv) were treated with prorenin (10–50 nmol/L), and cell lysates were analyzed by real-time RT-PCR for NFκB1 and NFκBia. Data are mean ± SEM, * P<0.05 vs. control, n = 5 each group. (**B**) Representative images of NFκB immunoreactivity and Dapi nuclear stain in N-9 (i) and primary microglial cell (ii) 24 hr post control (upper panels) and prorenin (20 nmol/L, lower panels) treatments. (**C**) N-9 (i–ii) and primary microglia (iii–iv) were treated with control solution (DMEM) or prorenin (20 nmol/L) in the presence or absence of PDTC (50 µmol/L), and IL-1β and TNFα mRNA levels were quantified by real-time RT-PCR. Experiments were performed triplicate. Data are mean ± SEM, * P<0.05 vs. control, n = 5 each group.

To determine if prorenin/PRR-elicited pro-inflammatory cytokine production is NFκB-dependent, we examined the expression of mRNAs for the subunits of the NFκB complex, NFκB1 and NFκBia mRNA, in both N-9 and primary microglial cells. The results demonstrated that prorenin induced a dose-dependent increase in both subunits (Figure 6A). Moreover, prorenin (20 nmol/L) promoted nuclear translocation of NFκB immunoreactivity (Figure 6B), suggesting that activation of NFκB is involved in prorenin-induced transcription. In order to substantiate the role of NFκB in this process, we treated both N-9 and primary microglia with prorenin (20 nmol/L) in the presence or absence of the NFκB inhibitor PDTC (50 µmol/L). The data shown in Figure 6C indicates that PDTC fully abolished the increases in cytokine mRNAs in both cell types, suggesting that the cytokine upregulation by prorenin/PRR is dependent on NFκB complex activation.

### Ang II potentiates prorenin-induced cytokine production in microglia

**Figure 7:**
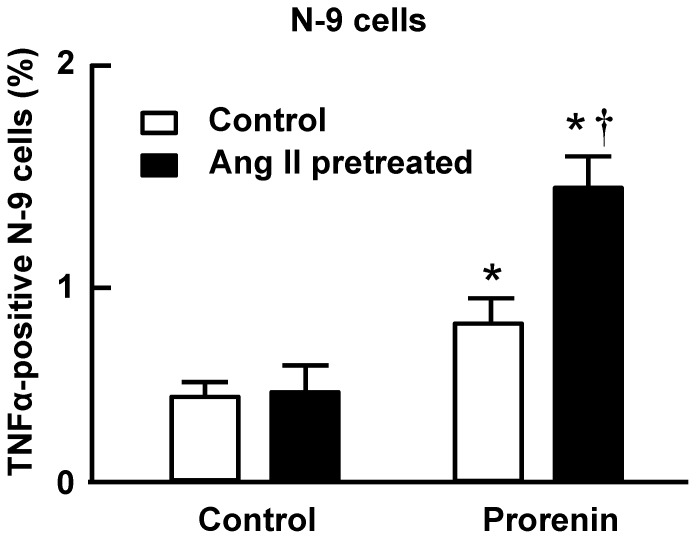
Ang II potentiates prorenin-induced TNFα production in microglia. Mouse N-9 cells were pretreated with control media (DMEM) or Ang II (100 nmol/L) for 12 h, followed by treatment with DMEM or prorenin (20 nmol/L) for 6 hr. Next, TNFα positive cells were analyzed by flow cytometry. Data are means ± SEM from n = 6 experiments. * P<0.05 vs. control; † P<0.05 vs. prorenin without Ang II pretreatment.

Considering that Ang II is a primary effector of the RAS, we tested whether Ang II and prorenin exert any synergistic actions. Flow cytometry analyses revealed that simultaneous co-treatment of N-9 cells with Ang II (100 nmol/L) and prorenin (20 nmol/L) for 6 hr produced no greater increase in TNFα protein levels when compared with prorenin alone (data not shown). Interestingly, after *pre-treatment* of N-9 cells with Ang II (100 nmol/L, 12 hr) the stimulatory effect of prorenin on TNFα levels was significantly enhanced versus that obtained in cells that had been pre-treated for 12 hr with control solution (Figure 7).

### Minocycline abolishes prorenin-induced increases in cytokine production

**Figure 8:**
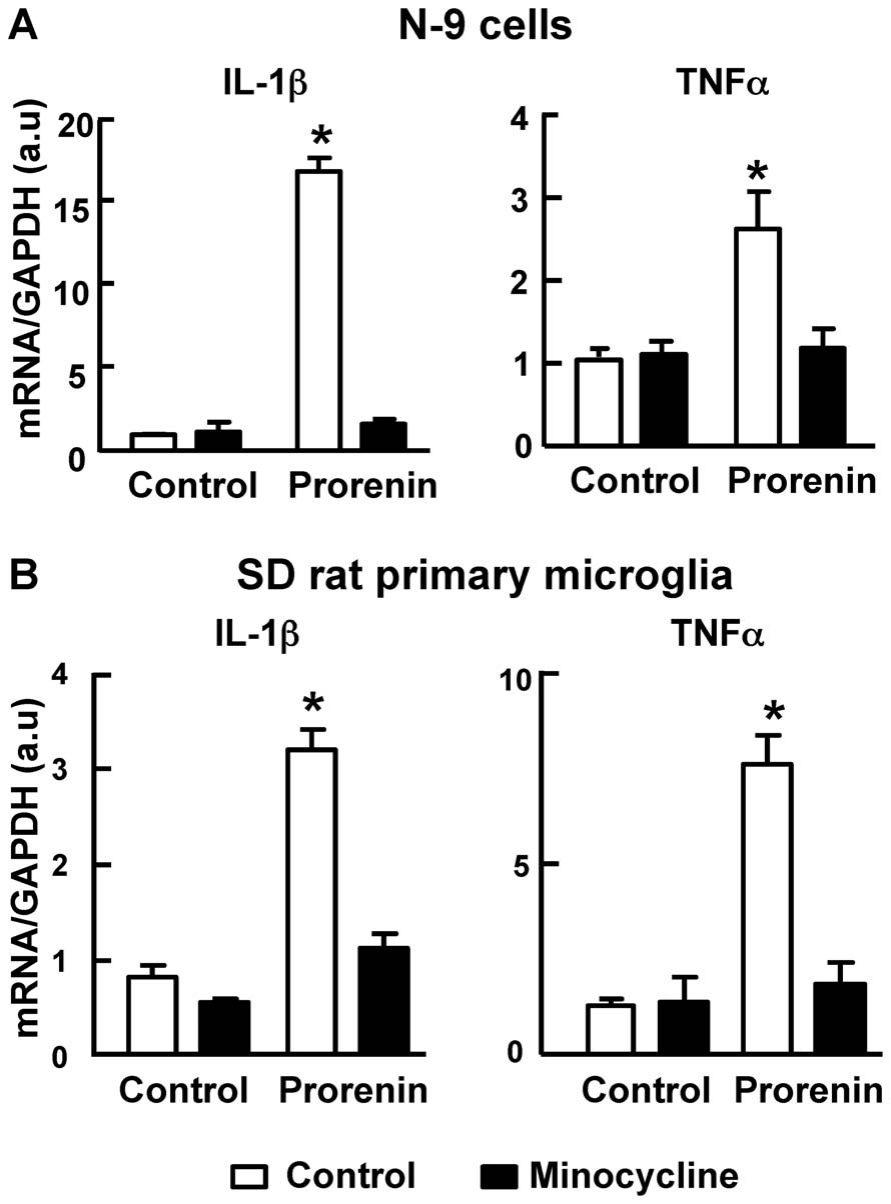
Minocycline inhibits prorenin induced pro-inflammatory cytokine production in microglial cells. Mouse N-9 (**A**) and SD rat primary microglial cells (**B**) were treated with control solution (DMEM) or prorenin (20 nmol/L) in the presence or absence of minocycline (1 µmol/L) for 24 hr; cell lysates were analyzed for IL-1β and TNFα mRNAs. Data are means ± SEM from 6 experiments. * P<0.05 vs. control.

In order to investigate whether the prorenin-induced increases in pro-inflammatory cytokine levels depends on microglial activation, we examined the mRNA expression of IL-1β and TNFα elicited by prorenin in the presence of minocycline, a potent inhibitor of microglial activation. Co-treatment of N-9 cells or primary microglia with minocycline (1 µmol/L) completely abolished the prorenin (20 nmol/L) induced increases in these cytokine mRNAs (Figure 8).

## Discussion

The major findings of the present study are: (i) that two rodent models which display neurogenic hypertension and over activation of the RAS in the brain (sRA mice and SHR) exhibit microglial activation, and increased levels of pro-inflammatory cytokines in the PVN; (ii) that the RAS component prorenin elicits *direct* activation of microglia and induction of pro-inflammatory mechanisms, effects that involve PRR-induced NFκB activation; (iii) that prorenin causes greater responses in cytokine production in Ang II-pretreated microglia; and (iv) prorenin-elicited increases in cytokine expression were fully abolished by microglial inhibitor minocycline. Taken together with our previous data which indicate that pro-inflammatory processes in the PVN are involved in the hypertensive action of RAS [12], the novel discovery that prorenin exerts direct stimulatory effects on microglial activation and pro-inflammatory cytokine production provides support for the idea that RAS-induced neurogenic hypertension is not restricted to actions of Ang II alone.

The present work raises many questions that will require further studies to answer. First of all, our in vitro studies suggest that the direct pro-inflammatory effects of prorenin in microglia are mediated through PRR, since they were not affected by the AT1R antagonist losartan and were attenuated/blocked by the putative PRR blocker HRP [27]. When considering the debatable specificity of HRP in blocking PRR, an alternative approach to address whether prorenin induced pro-inflammatory effects are mediated by PRR would be to down-regulate PRR in the microglia and then examine the effects on inflammation formation. While our *in vitro* data suggest a direct induction of pro-inflammatory cytokines by prorenin, it is likely that in the *in vivo* situation prorenin also operates indirectly via the generation of Ang II.

Our studies also indicated that while the pro-inflammatory actions of prorenin in microglia were AT1R-independent, they were potentiated by pretreatment of the cultures with Ang II (Figure 7). This “priming” action of Ang II appears similar to that of interferon gamma (IFNγ), a physiological activator of macrophages. By itself, IFNγ has limited effects on cytokine production. However, it primes the cells to produce a much stronger effects to a second stimulation, such as elicited by LPS, Cytosine-phosphate-guanosine (CpG) and TNFα [28]–[30]. A possible explanation is that pretreatment of microglia with Ang II modulates an intracellular event that potentiates the pro-inflammatory effects of prorenin. Our data (Figure 6) indicate that the pro-inflammatory action of prorenin action is involves an NFκB-dependent mechanism. Thus, it will be interesting to determine whether the priming actions of Ang II also involve an NFκB-dependent mechanism, or a mechanism that is independent of that activated by prorenin.

An important question raised by these studies is whether the pro-inflammatory cytokines that are induced by RAS activation exert stimulatory effects on sympathetic outflow. We can speculate that pro-inflammatory cytokines, or other microglial-derived factors such as chemokines, elicit sustained increases in the activity of PVN pre-sympathetic neurons that *enhance* the direct neuronal actions of Ang II via AT1R to produce chronic sympathoexcitation. However, while studies have demonstrated cardiovascular actions of cytokines injected into the PVN [12], [31], and it has been demonstrated that IL-1β depolarizes parvocellular neurons at this site [32], the presence of pro-inflammatory cytokine receptors on pre-autonomic efferent neurons in the PVN has not been demonstrated. In fact, one study has demonstrated the presence of IL-1β receptors on magnocellular secretory neurons at this site [33]. Thus, it is possible that pro-inflammatory cytokines, induced by RAS activation, have no direct stimulatory effects on pre-sympathetic neurons but operate to enhance sympathoexcitation via effects on interneurons or by inhibition of GABAergic input, as is the case in the aforementioned depolarization of PVN neurons by IL-1β [32]. A major goal of our current and future research will be to examine whether there is a direct linkage between prorenin-induced pro-inflammatory actions in the PVN and activation of pre-sympathetic neurons at that site.

The current studies were focused on microglia, the major resident immune cells in the brain, because of our previous observation that inhibition of microglial activation by minocycline treatment blunted Ang II-induced hypertension. However, it is likely that other immune cells may also contribute to RAS-induced neuroinflammation. For example, it is well known that astrocytes are also a major contributor to resident immune mechanisms in the central nervous system [34], and contain both AT1R and PRR [20], [35]. In addition, perivascular macrophages have been shown to be another source of inflammatory mediators in the CNS, and factors released from these cells such as cyclooxygenase 2 and prostaglandin E2 can infiltrate the blood-brain barrier and increase neuronal activity in the PVN [36]. Since microglia are resident immune cells and it is very likely that they are one of the earliest players, and might recruit patrolling perivascular macrophages to exaggerate the inflammatory reactions with the progress of hypertension. Therefore, the present study does not exclude the possibility that perivascular macrophages contribute to the RAS-dependent inflammation. A further possibility is that prorenin induced activation of microglia functions as an initiating event, recruiting and activating the adaptive immune system such as circulating T lymphocytes [37] or monocytes [38] to the PVN to exacerbate the immune reaction. Clearly, further studies/experiments need to be performed to determine the contribution from individual immune cell populations.

In conclusion, the data presented here provide evidence for *direct* actions of prorenin at microglia to exert pro-inflammatory actions. When considering that animal models of neurogenic hypertension display increased RAS activation in the PVN and also increased activation of microglia and levels of pro-inflammatory mediators at this site, our findings support the notion that the hypertensive action of prorenin via the brain includes a neuroimmune component at the PVN.
